# Evaluating the use of laparoscopic gastrostomy in children with congenital heart disease in Colombia: a retrospective analysis

**DOI:** 10.3389/fped.2024.1405793

**Published:** 2024-06-13

**Authors:** Diego Fernando Chaparro-Zaraza, Adriana Patricia Pinilla-Orejarena, Juan Pablo Otoya-Castrillón, Daniella Chacón-Valenzuela, Juan Jose Quintero-Olarte, Angélica Belen Cifuentes-Rincón, Bryan Felipe Quesada-Uribe, Alvaro Durán-Hernandez, Anderson Bermon, Edgar Fabian Manrique-Hernandez

**Affiliations:** ^1^ Department of Surgery, Hospital Internacional de Colombia, Piedecuesta, Colombia; ^2^ Department of Pediatric Surgery, Fundación Cardiovascular de Colombia, Floridablanca, Colombia; ^3^ School of Medicine, Universidad Industrial de Santander, Bucaramanga, Colombia; ^4^ Department of Pediatric Intensive Care, Fundación Cardiovascular de Colombia, Floridablanca, Colombia; ^5^ Department of Epidemiology, Fundación Cardiovascular de Colombia, Floridablanca, Colombia

**Keywords:** congenital heart disease, gastrostomy, laparoscopy, perioperative complication, outcomes

## Abstract

**Introduction:**

Congenital Heart Disease (CHD) is the most common congenital disorder and a leading cause of infant mortality. Despite improved survival rates, patients with CHD often face malnutrition due to increased metabolic demands, feeding difficulties, and gastrointestinal dysfunction. Malnutrition in CHD is linked to poor short and long-term clinical outcomes. Gastrostomy (GT) is frequently used for long-term enteral support, and laparoscopic GT (LGT) has demonstrated advantages in children without CHD. This study evaluated a modified Georgeson's percutaneous LGT technique and its perioperative complications in children with CHD.

**Methods:**

We performed an analytical retrospective cohort study from 2018 to 2022, including patients younger than 24 months with a diagnosis of CHD who underwent LGT. The primary outcome evaluated was the presence of complications during surgery and the first thirty postoperative days. Complications were graded using Clavien–Dindo's (CD) classification. Sociodemographic, clinical, and procedure-related variables were collected. A bivariate analysis was performed using STATA 15, and a *p* < 0.05 was considered statistically significant.

**Results:**

Seventy-eight patients were eligible (male 56.41%, Median age 129.5 days, weight: 4.83 kg). The median surgery time was 35 min. The complication rate was 24.36%. The most frequent complications were GT site infection (10.26%), followed by leakage (8.97%) and granuloma formation (6.41%). Conversion to open surgery was significantly associated with postoperative complications (*p* = 0.002).

**Conclusion:**

This modified technique is well-tolerated in children with CHD, demonstrating a low rate of CD grade 3A/3B complications and no grade 4 or 5 complications.

## 1 Introduction

Congenital Heart Disease (CHD) is the most common type of congenital disorder and a leading cause of infant mortality worldwide (1). Medical advancements have significantly improved the survival rates of children with CHD. However, these patients often face complex issues, such as malnutrition and failure to thrive (2–4). It has been reported that children under two years of age with CHD have a 51.2% and 40.5% prevalence of acute and chronic malnutrition, respectively (5). Proper nutrition in these patients can be impaired due to a combination of factors, including increased metabolic demands, feeding difficulties, and gastrointestinal dysfunction (3). The increased metabolic demands are mainly due to the higher energy expenditure required to maintain cardiac output and support growth, especially in those who undergo the stress of cardiac surgery or are critically ill (3–5). Feeding difficulties in these patients can be attributed to associated malformations, comorbidities, and swallowing disorders secondary to vocal cord paralysis due to incidental recurrent laryngeal nerve lesions during cardiac surgery (3, 4).

Perioperative malnutrition and impaired growth in patients with CHD are associated with poor short and long-term clinical outcomes. Previous studies have shown an increased risk of infection, more extended hospital stays, prolonged need for respiratory support, higher risk of all-cause mortality, and a delay in neurodevelopment (3, 5–8). Despite this, there is no consensus on optimal feeding practices for children with CHD, and the management of these patients varies across institutions (3, 9). The clinical team should adjust the nutritional support to the patient's CHD type and surgical period; enteral nutrition should be initiated as early as possible and complemented with parenteral nutrition whenever children cannot tolerate adequate enteral feeding to meet their caloric needs (9).

Placing a gastrostomy (GT) is one of the strategies available for patients who cannot tolerate oral feedings due to a high risk of aspiration and who require long-term enteral nutritional support. Previous studies in children without CHD demonstrated that laparoscopic gastrostomy (LGT) has multiple advantages over other techniques: a lower rate of major complications compared to the endoscopic and percutaneous approach and a shorter hospital stay compared to open surgery (10). Since children with CHD have a higher surgical risk compared to those without CHD, surgeons should utilize the safest method for GT placement. Reports also state that laparoscopic surgery can be safely performed even in patients with single-ventricle physiology (11). Nevertheless, Open GT placement continues to be used more often in this group of patients (12).

Only a few studies have tried to establish which method of GT placement is associated with better outcomes in children with CHD; there has yet to be a consensus (12). Additionally, various LGT techniques exist, and it is unclear whether any is superior or what factors are associated with complications. This study aimed to evaluate a modification of Georgeson's percutaneous LGT technique in children with CHD and its perioperative complications.

## 2 Materials and methods

### 2.1 Study design

**Figure 1:**
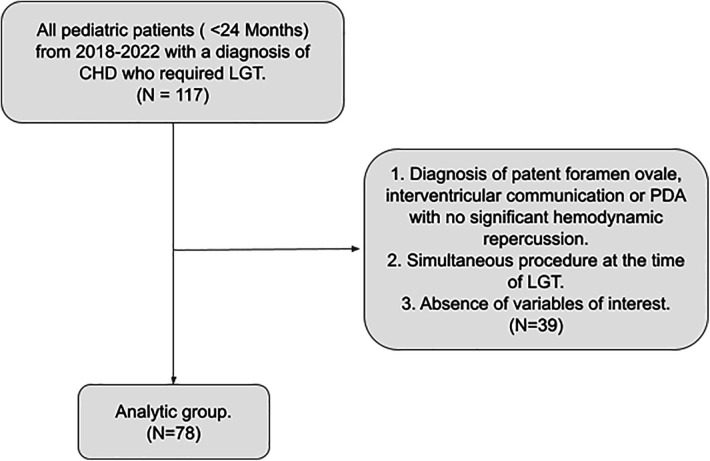
Flowchart summarizing the selection process of patients with congenital heart disease who underwent laparoscopic gastrostomy included in our study.

Following approval of the Research Ethics Committee (CEI-2023-06054), an analytical retrospective cohort study was performed, including patients younger than 24 months with an echocardiographic diagnosis of CHD who underwent LGT at a reference cardiovascular center in the northeastern region of Colombia from 2018 to 2022. To eliminate potential confounders, we excluded patients undergoing simultaneous surgical procedures with GT and those lacking variables of interest in their medical records (Figure 1).

Statistical analyses were performed using STATA15. Categorical variables were presented as absolute frequencies and percentages. Normality was assessed using the Shapiro–Wilk test for continuous variables. Normally distributed variables were reported with mean and standard deviation, while non-normally distributed variables were reported with medians and interquartile ranges (IQR). The bivariate analysis used the chi-square and Fisher's exact tests for categorical variables. The t-test was used for continuous variables with normal distribution, and the Mann–Whitney *U* test was used for non-normally distributed variables. Kaplan–Meier Curves were constructed in patients with and without perioperative complications, having as variables (time to event): age in days at the time of LGT, duration in minutes of LGT, hospitalization duration in days at the time of LGT, and total hospital stay duration in days. A *p* < 0.05 was defined as the cutoff for statistical significance.

### 2.2 Primary outcome and other variables

The primary outcome evaluated was the presence of LGT-related complications during surgery and the first 30 postoperative days. Postoperative complications were defined as displacement of the GT button, leakage, infection, bleeding, granuloma formation, incorrect positioning, stoma dilation, dislodgement of the GT, and death. Perioperative complications recorded were graded according to the Clavien–Dindo (CD) classiﬁcation.

The analysis included demographic variables, comorbidities, type of CHD, history of cardiovascular surgery before the procedure, primary indication for GT, time of hospital stay, hospitalization days at the time of the GT, preoperative nutrition type, duration of surgery, number of trocars used, participation of a cardiovascular anesthesiologist during the procedure, days of postoperative fasting, days of gastropexy suture removal, and education provided to the patient's family before hospital discharge.

### 2.3 Surgical indication and technique

In our institution, oral nutrition is prioritized whenever possible. If a patient has a contraindication for oral feeding, gastric nutrition via nasogastric or nasojejunal feeding tube is preferred. Those who exhibit intolerance to enteral feeding receive parenteral nutrition. Every patient with a swallowing disorder undergoes speech therapy rehabilitation for two weeks. After this period, a videofluoroscopic swallowing study is performed. If the study results are unsatisfactory but the child shows clinical improvement, two additional weeks of speech therapy are provided. Should the risk of aspiration persist, the option of a GT feeding tube is considered. Additionally, a nutritional support team assesses all patients, and the nutritional formula they receive through the nasogastric tube is tailored to their requirements.

**Figure 2:**
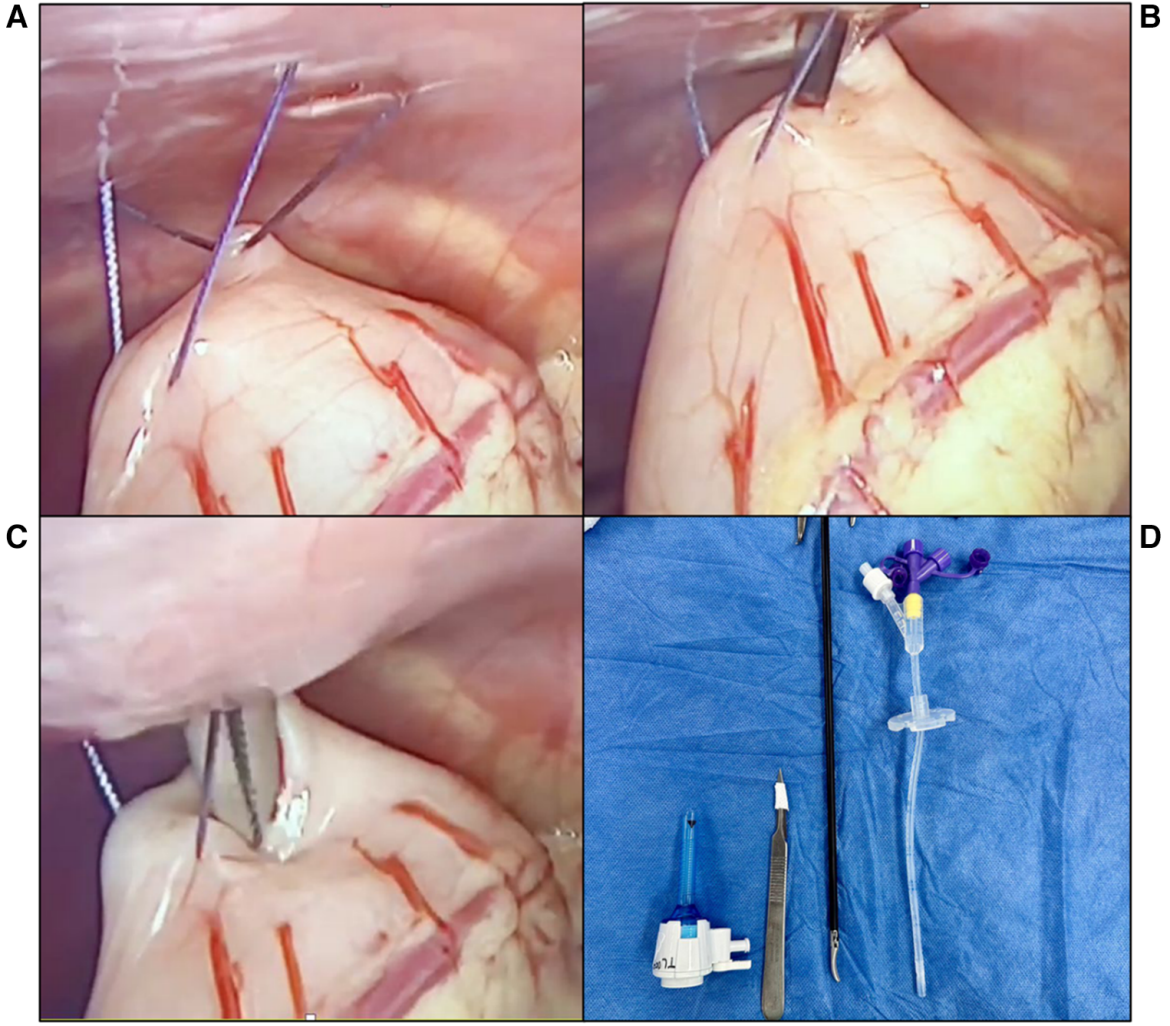
Steps for the percutaneous laparoscopic gastrostomy technique used in our center. (**A**) Trans-parietal U stitches for gastropexy using 2-0 polyglactin. (**B**) 5 mm Incision on the anterior gastric wall performed using an #11 blade. (**C**) Gentle dilation of the incision using straight mosquito forceps. (**D**) Instruments used for this LGT technique.

We do not utilize Seldinger's catheter insertion technique for the GT placement because these LGT insertion kits are unavailable. Since 2016, we have been using a modification of Georgeson's LGT. With routine administration of prophylactic antibiotics and under general anesthesia, a 5 mm trocar is inserted through the umbilicus using an open technique. The pneumoperitoneum is established with an approximate pressure of 10 mmHg and a flow of 3–5 L/min. A 30-degree lens is inserted to visualize the stomach and abdominal cavity. Subsequently, a small incision is made in the left hypochondrium through which Maryland forceps are inserted, and the anterior wall of the stomach at the body-antrum junction is retracted. If the stomach is not easily visualized, the anesthesiologist is asked to insufflate between 20 and 60 ccs of air through a nasogastric tube. Two trans-parietal U stitches of gastropexy are placed using 2-0 polyglactin to anchor the stomach to the abdominal wall temporarily. In the middle of these stitches, a gastrotomy of approximately 5 mm is performed using a #11 blade and is gently dilated with straight mosquito forceps. The GT tube is inserted, and the balloon is inflated with four ccs of water. For infants six months or younger, we utilize a 12 French tube, and for those older than six, a 14 French tube. Afterward, the intragastric position of the balloon is verified, and the retainer is fixed to the abdominal wall by knotting the trans-parietal gastropexy stitches outside. Finally, the fascia and skin are closed using absorbable sutures (Figure 2).

## 3 Results

### 3.1 Patient and procedure characteristics

Seventy-eight of one hundred and seventeen patients with CHD who underwent LGT during the study period met the inclusion criteria (Figure 1). Most of them were male (56.41%) and born at term (65.38%. At the time of the procedure, the median age was 129.5 days (IQR: 89–185), and the median weight was 4.83 kg (IQR: 3.9–5.6). Around two-thirds (62.34%) of the patients presented a non-cyanotic CHD. The most frequent type of CHD was the ventricular septal defect (17.95%), followed by aortic coarctation (15.38%) and patent ductus arteriosus (PDA) (14.10%). The majority of patients presented a prior neurological abnormality (65.38%), and the primary indication for GT was the suction-swallowing disorder (64.10%). For most patients (83.33%), cardiac surgery was performed before LGT placement. (Table 1). Before cardiac surgery, most of the children with CHD received total parenteral nutrition (46.15%), followed by enteral (21.79%) and oral nutrition (21.79%).

**Table 1:** Sociodemographic, clinical and procedure characteristics of children with a diagnosis of congenital heart disease undergoing laparoscopic gastrostomy.

Variable	Category	*n* = 78 (%)
Sex	Female	34 (43.59)
	Male	44 (56.41)
Gestational age	Pre-term	27 (34.62)
	Full-term	51 (65.38)
Age at GT, days	Median—IQR	129.5 (89–185)
Weight at GT, Kg	Median—IQR	4.83 (3.9–5.6)
CHD type	Cyanotic	29 (37.66)
	Non cyanotic	48 (62.34)
First procedure	Cardiac	65 (83.33)
	Gastrostomy	13 (16.67)
Previous neurological impairment		51 (65.38)
GT indication	Sucking/suction disorder	50 (64.10)
	Neurogenic dysphagia	15 (19.23)
	Vocal cord paralysis	10 (12.82)
	Prolonged nasogastric tube feeding (>4 weeks).	2 (2.56)
	Other	1 (1.28)
Amount of trocars used in GT	1	75 (96.15)
	2	2 (2.56)
	3	1 (1.28)
Conversion to open surgery		3 (3.85)
Need for surgical reintervention		3 (3.85)

CHD, congenital heart defect; LGT, laparoscopic gastrostomy; GT, gastrostomy.

The median total hospital stay was 97.5 days (IQR: 59–150), and the median hospitalization at the time of the procedure was 80.5 days (IQR: 47–120). The median duration of the surgical procedure was 32 min (IQR: 28–43). Three patients (3.85%) required conversion of the procedure to open surgery: one case due to microgastria that limited stomach mobilization and the other two due to impaired visualization of the abdominal cavity (secondary to insufficient CO2/distention of intestinal loops & marked hepatomegaly). Patients were subject to postoperative fasting for a median of two days (IQR: 1–3), and gastropexy sutures were removed at a median of four days (IQR: 4–5). All patients (100%) received education from the ostomy care nursing team before discharge (Table 2).

**Table 2:** Factors associated with LGT related complications in children with CHD.

Variables	Categories	Complications	No complications	*P* value^a^
		19 (%)	59 (%)	
Sex	Female	6 (31.58)	28 (47.46)	0.225
	Male	13 (68.42)	31 (52.54)	
CHD type	Cyanotic	9 (47.37)	20 (33.9)	0.217
	Non cyanotic	10 (52.63)	39 (66.1)	
Corrected CHD	Yes	12 (63.16)	43 (72.88)	0.419
	No	7 (36.84)	16 (27.12)	
First procedure	Cardiac	15 (78.95)	50 (84.75)	0.555
	Gastrostomy	4 (21.05)	9 (15.25)	
GT Indication	Sucking/suction disorder	14 (73.68)	36 (61.02)	0.369
	Neurogenic dysphagia	2 (10.53)	13 (22.03)	
	Vocal cord paralysis	2 (10.539	8 (13.56)	
	Prolonged nasogastric tube feeding	0 (0)	2 (3.39)	
	Other	1 (5.26)	0 (0)	
Type of nutrition before cardiac surgery	Oral	3 (15.79)	9 (15.25)	0.515
	Enteral (Tube).	6 (31.58)	11 (45.76)	
	Total parenteral	9 (47.37)	27 (45.76)	
	Mixed	1 (5.26)	8 (13.56)	
	No cardiac Surgery	0 (0)	4 (6.78)	
Cardiovascular anesthesiologist	Yes	12 (63.16)	23 (38.98)	0.065
	No	7 (36.84	36 (61.02)	
Conversion to open surgery	Yes	3 (15.79)	0 (0)	0.002
	No	16 (84.21)	59 (100)	
Postoperative fasting	Yes	18 (94.74)	54 (91.53)	0.648
	No	1 (5.26)	5 (8.47)	
Reintervention	Yes	3 (15.79)	0 (0)	0.002
	No	16 (84.21)	59 (100)	
Hospital stay, days	Median (IQR)	120 (76–178)	94 (58–141)	0.188^b^
Hospitalization duration at the time of LGT, days	Median (IQR)	93 (57–120)	77 (47–121)	0.474^b^
Age at the time of LGT, days	Median (IQR)	127 (74–219)	139 (89–182)	0.373^b^
Duration of LGT, minutes	Median (IQR)	35 (27–55)	32 (28–40)	0.540^b^
Weight at surgery, Kg	Median (IQR)	4.7 (3.9–5.89)	4.84 (3.89–5.59)	0.852^b^

CHD, congenital Heart defect; LGT, laparoscopic gastrostomy; GT, gastrostomy; IQR, interquartile range.

^a^ By Chi-squared or Fisher's exact when applicable.

^b^ By Mann–Whitney *U* test.

### 3.2 Factors associated with perioperative complications

In total, nineteen patients (24.36%) presented with at least one perioperative complication. The majority of complications were CD grade 1 (10.26%) and grade 2 (8.97%), with fewer cases of grade 3A (1.28%) and grade 3B (3.85%). No CD grade 4 or 5 complications were documented. The most frequent complications were GT site infection (10.26%), followed by leakage (8.97%) and granuloma formation (6.41%) (Table 3).

**Table 3:** Perioperative (<30 days) complications distribution by Clavien–Dindo grade and type of complication rates.

Variable	Category	*n* = 78 (%)
Clavien–Dindo grade	1	8 (10.25)
	2	7 (8.97)
	3A	1 (1.28)
	3B	3 (3.85)
	4–5	0 (0)
Type of complication	Leakage	7 (8.97)
	GT button displacement	1 (1.28)
	GT site infection	8 (10.26)
	GT site bleeding	3 (3.85)
	Stoma granuloma formation	5 (6.41)
	Incorrect positioning	2 (2.56)
	Stoma dilation	4 (5.13)
	GT tube dislodgement	3 (3.84)

CHD, congenital heart defect; LGT, laparoscopic gastrostomy; GT, gastrostomy.

The only CD grade 3A complication occured on a two-month-old, 5 kg patient who had GT dislodgement due to the accidental connection of the enteral nutrition to the balloon port, leading to its partial deflation; this situation was managed by changing the GT tube for a new one and verifying the absence of leakage in the abdominal cavity with a contrasted radiographic study. The first case of CD grade 3B complication was a seven-month-old and 6 kg female patient who had a markedly enlarged liver during the initial LGT. In the postoperative period, the patient presented with stoma dilation, external leakage, peristomal cellulitis, and GT dysfunction. She received antibiotic therapy, and multiple non-invasive maneuvers were attempted, but eventually required a GT remodeling. The second case was a two-month-old 3.9 kg male patient who presented stoma dilation and signs of abdominal irritation a day after the initial LGT; he was taken to surgery, where severe hepatomegaly that extended to the left iliac fossa and pressed over the stomach dislodging the GT was evidenced. This case required gastrorrhaphy and a new GT using Stamm's open technique. The last CD grade 3B case was a two-month-old 3.8 kg male patient who presented with peritonitis secondary to GT dislodgement and required a new open surgical intervention with gastrorrhaphy and new GT placement.

Among patients that presented complications, the most common CHD was the Tetralogy of Fallot (21.05%), followed by Transposition of the Great Vessels (15.79%) and PDA (15.79%). Complications occurred more frequently in male patients (68.42%) and in those with non-cyanotic CHD (52.63%). All patients who required conversion of the procedure to open surgery presented some perioperative complication (*P* = 0.002) (Table 2).

Most cases that presented complications underwent cardiac surgery (either palliative or corrective) before GT placement (78.95%). Most of them happened when the indication for GT was a sucking and swallowing disorder (73.68%). Patients with perioperative complications mostly received non-oral feeding, with total parenteral nutrition being the most frequent (47.37%). However, these variables showed no statistically significant difference between complicated and uncomplicated patients (Table 2).

**Figure 3:**
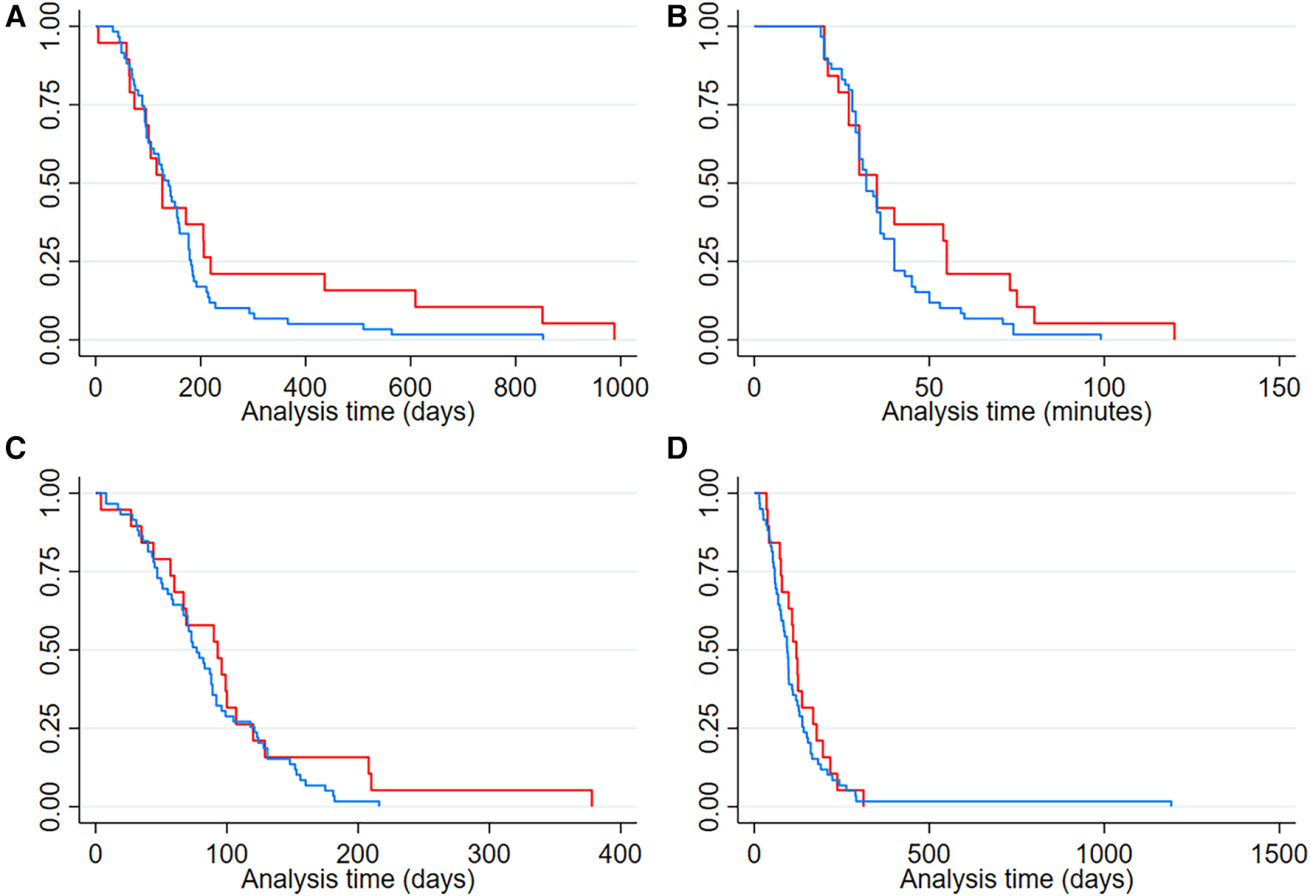
Kaplan–Meier curves showing time differences in patients with and without perioperative complications (**A**) Age in days at the time of laparoscopic gastrostomy (**B**) duration in minutes of laparoscopic gastrostomy (**C**) hospitalization duration in days at the time of laparoscopic gastrostomy (**D**) hospital stay duration in days. Red line: Patients with postoperative complications. Blue line: Patients without postoperative complications.

Patients that presented complications had a longer hospitalization duration at the time of the procedure (Median = 93 days, IQR: 57–120), were younger at the time of the surgery (Median = 127 days, IQR: 74–219), had a longer procedure time (Median = 35 min, IQR: 27–55) and a more extended hospital stay (Median = 120 days, IQR: 76–178). However, these differences did not reach statistical significance (See Table 2; Figure 3 for more details).

## 4 Discussion

This study presented our experience using a modified LGT technique in seventy-eight children with CHD. To date, this is the first analytical research conducted in Latin America that evaluates the factors associated with perioperative complications of LGT in this patient group. Our approach yielded a 24.36% complication rate, with the most common complications being GT site infection, leakage, and granuloma formation. It also identified a significant association between the conversion of the procedure to open surgery and postoperative complications (*P* = 0.002).

There is a single descriptive study with a population demographically comparable to ours published by Salamanca et al. (2017), in which a series of 47 Colombian children with CHD were operated using the mini-stoma laparoscopic technique. In this study, the majority of patients were male (66%), had a Median age of 6 months, presented with ventricular septal defects (17%), were diagnosed with sucking/swallowing disorder (93%), and had a pre-existing neurological involvement (85%), similar to the findings of our study. However, the rate of postoperative complications was higher than ours (40%), and the most frequent complication was GT leakage (19.14%), which is inconsistent with our findings (13). These differences could be due to the different laparoscopic techniques or other unexplored variables.

In the international literature, the rate of complications in patients undergoing LGT varies widely, ranging from 13.8% to 69% (12–18). It is important to emphasize that in some of the available studies, the follow-up period extended up to one-year post-GT, while in others, it was not even specified, which may explain the variability in the reported rates. Moreover, the selection criteria for patients, such as age, the procedure performed, and types of CHD, are highly heterogeneous among the available studies, making it challenging to compare them (14–18). The paper by Norén et al. is the only one exclusively including children with CHD who underwent LGT using a modified Stamm technique, and in this study, the complication rate was 69% (14).

An incongruity in available data regarding the most frequent postoperative complication is also noticeable. Studies report granulation tissue formation as one of the main complications, with a rate of up to 40% (15–18). Our findings are consistent with other studies indicating that stoma infection is the primary complication of LGT (12,14–6,18). In the study by Noren et al., the order of the main complications was as follows: stoma infection (48%), followed by leakage (41.3%), and granuloma formation (41.3%), which aligns with the results of our research (14).

Previous studies have compared patients based on the classification of the heart defect (Univentricular/Biventricular and Cyanotic/Non-cyanotic) to analyze how CHD relates to postoperative complications (14, 15). So far, no study has found statistically significant differences in the frequency and type of complications presented between these groups, which is consistent with what is reported in this article.

Furthermore, there is scarce data regarding other factors associated with postoperative complications in patients with CHD undergoing LGT. In their study, Tran et al. reported that receiving oral feeding before cardiovascular intervention and discharging the patient within the first two weeks after GT were protective factors (16). Although differences between patients with and without complications were observed, they were not statistically significant.

This LGT technique did not report any hemodynamic complications related to pneumoperitoneum during GT, and there was no transoperative mortality. These findings support previous evidence stating that children with CHD can safely undergo laparoscopic surgery (11). Also, when analyzing whether the participation of a cardiovascular anesthesiologist in the surgical case was associated with an improvement in postoperative outcomes, no statistically significant differences were found. The previous suggests that this procedure could be carried out even in centers that do not specialize in cardiac patients.

Most of the studies available utilize the laparoscopic Stamm GT technique or the percutaneous laparoscopic technique described by Georgeson, in which sutures that traverse the abdominal wall facilitate percutaneous access to the GT site using serial dilations with the standard Seldinger technique (14, 19, 20). We believe that instead of advancing a guidewire with serial dilators (10, 16, and 20 Fr), performing the dilation using instruments readily available allows for a short surgery time (Median = 32 min) without increasing the rate of leakage (8.97%) and other complications. Additionally, at our institution, medication administration and enteral stimulation through the GT are initiated 24 h postoperatively, and the gastropexy stitches are removed on the fourth postoperative day, as in our experience, leaving them longer seems to irritate the surrounding skin at the stoma and increase granuloma formation. Our patient's families must receive education sessions that the ostomy care nursing team imparts during hospitalization and before discharge, which might positively impact the rate of postoperative complications related to GT.

It is essential to acknowledge the limitations of this study. First, the retrospective design makes it subject to selection bias and dependent on the accuracy and completeness of data available in medical records. Nevertheless, the variables of interest were readily available for analysis due to our systematic documentation practices during patient follow-up. Also, patients whose medical history lacked the information necessary for our analysis were excluded. Second, this is a single-center study in a fourth-level institution that cares for critically ill children with CHD, which may constrain our findings’ external validity or generalizability. Third, the follow-up period was limited, focusing exclusively on postoperative complications occurring within the initial 30 days following LGT placement, which might limit comparison with other studies. Despite these limitations, this research significantly contributes to the existing body of evidence. Its results provide valuable insights into this topic and establish a foundation for further discussion in countries across Latin America. Further studies are needed to validate our findings in more extensive and diverse groups, explore additional factors related to postoperative complications, and compare the outcomes of various existing LGT techniques.

To conclude, the modified LGT technique used in our institutions is notably well-tolerated in children with CHD since it did not cause any hemodynamic repercussions related to pneumoperitoneum or transoperative mortality. Additionally, this modification had a low CD grade 3A and 3B complications rate and did not yield any grade 4 or 5 complications. This study’s results show that conversion to open surgery is significantly associated with postoperative complications in LGT. We plan to look further into how different CHDs affect LGT outcomes and report longer follow-up data on patients who have been operated on using this technique.

## Data Availability

The raw data supporting the conclusions of this article will be made available by the authors, without undue reservation.

## References

[1] Tennant PW Pearce MS Bythell M Rankin J. 20-year survival of children born with congenital anomalies: a population-based study. Lancet. (2010) 375(9715):649–56. 10.1016/S0140-6736(09)61922-X 20092884

[2] Tsintoni A Dimitriou G Karatza AA. Nutrition of neonates with congenital heart disease: existing evidence, conflicts and concerns. J Matern Fetal Neonatal Med. (2020) 33(14):2487–92. 10.1080/14767058.2018.1548602 30608033

[3] Medoff-Cooper B Ravishankar C. Nutrition and growth in congenital heart disease: a challenge in children. Curr Opin Cardiol. (2013) 28(2):122–9. 10.1097/HCO.0b013e32835dd005 23370229

[4] Mangili G Garzoli E Sadou Y. Feeding dysfunctions and failure to thrive in neonates with congenital heart diseases. Pediatr Med Chir. (2018) 40(1):1–2. 10.4081/pmc.2018.196 29871471

[5] Toole BJ Toole LE Kyle UG Cabrera AG Orellana RA Coss-Bu JA. Perioperative nutritional support and malnutrition in infants and children with congenital heart disease. Congenit Heart Dis. (2014) 9(1):15–25. 10.1111/chd.12064 23602045

[6] Mitting R Marino L Macrae D Shastri N Meyer R Pathan N. Nutritional status and clinical outcome in postterm neonates undergoing surgery for congenital heart disease. Pediatr Crit Care Med. (2015) 16(5):448–52. 10.1097/PCC.0000000000000402 25828781

[7] Ravishankar C Zak V Williams IA Bellinger DC Gaynor JW Ghanayem NS Pediatric heart network investigators. Association of impaired linear growth and worse neurodevelopmental outcome in infants with single ventricle physiology: a report from the pediatric heart network infant single ventricle trial. J Pediatr. (2013) 162(2):250–6.e2. 10.1016/j.jpeds.2012.07.048 22939929 PMC3547153

[8] Eskedal LT Hagemo PS Seem E Eskild A Cvancarova M Seiler S Impaired weight gain predicts risk of late death after surgery for congenital heart defects. Arch Dis Child. (2008) 93(6):495–501. 10.1136/adc.2007.126219 18230653

[9] Kataria-Hale J Gollins L Bonagurio K Blanco C Hair AB. Nutrition for infants with congenital heart disease. Clin Perinatol. (2023) 50(3):699–713. 10.1016/j.clp.2023.04.007 37536773

[10] Baker L Beres AL Baird R. A systematic review and meta-analysis of gastrostomy insertion techniques in children. J Pediatr Surg. (2015) 50(5):718–25. 10.1016/j.jpedsurg.2015.02.021 25783383

[11] Gillory LA Megison ML Harmon CM Chen MK Anderson S Chong AJ Laparoscopic surgery in children with congenital heart disease. J Pediatr Surg. (2012) 47(6):1084–8. 10.1016/j.jpedsurg.2012.03.008 22703774

[12] Huerta CT Ramsey WA Courel SC Saberi RA Gilna GP Ribieras AJ Outcomes of gastrostomy tubes in newborns with congenital heart disease and comparison of techniques. J Surg Res. (2022) 280:475–85. 10.1016/j.jss.2022.07.028 36063624

[13] Salamanca E Sebá JE Suárez A. Experiencia en niños de la gastrostomía laparoscópica por miniestoma. Rev Colomb Cir. (2017) 32:176–81. 10.30944/20117582.22

[14] Norén E Gunnarsdóttir A Hanséus K Arnbjörnsson E. Laparoscopic gastrostomy in children with congenital heart disease. J Laparoendosc Adv Surg Tech A. (2007) 17(4):483–9. 10.1089/lap.2006.0119 17705732

[15] Shahi N Phillips R Meier M Shirek G Goldsmith A Soden JS Gastrostomy button placement in infants with cyanotic versus acyanotic congenital heart disease. J Surg Res. (2021) 259:407–13. 10.1016/j.jss.2020.09.014 33616074

[16] Tran NN Mahdi EM Ourshalimian S Sanborn S Alquiros MT Kingston P Factors associated with gastrostomy tube complications in infants with congenital heart disease. J Surg Res. (2022) 280:273–9. 10.1016/j.jss.2022.07.022 36030602 PMC10231870

[17] Jensen AR Rao R Herrmann JL Markel TA Gray BW. Surgical gastrostomy in pediatric patients undergoing cardiac surgery. J Surg Res. (2021) 259:516–22. 10.1016/j.jss.2020.10.004 33218701

[18] Ascencio A Fingland S Diaz-Miron J Weber N Hills-Dunlap J Partrick D Operative complications following gastrostomy tube placement after cardiac surgery during infancy. J Surg Res. (2024) 296:203–8. 10.1016/j.jss.2023.12.030 38281355

[19] Akay B Capizzani TR Lee AM Drongowski RA Geiger JD Hirschl RB Gastrostomy tube placement in infants and children: is there a preferred technique? J Pediatr Surg. (2010) 45(6):1147–52. 10.1016/j.jpedsurg.2010.02.079 20620310

[20] Casar Berazaluce AM Garrison AP Ponsky TA. Gastrostomy. In: Davenport M Geiger JD editors. Operative Pediatric Surgery. 8th ed. Florida: CRC Press (2021). p. 808–12.

